# Expression of pyrimidine nucleoside phosphorylase mRNA plays an important role in the prognosis of patients with oesophageal cancer

**DOI:** 10.1038/sj.bjc.6690089

**Published:** 1999-02

**Authors:** M Yamagata, M Mori, K Mimori, K-I Mafune, Y Tanaka, H Ueo, T Akiyoshi

**Affiliations:** Department of Surgery, Medical Institute of Bioregulation, Kyushu University, Beppu, Japan; Department of Surgery, Tokyo University Hospital, Tokyo, Japan; Department of Surgery, Suita Cancer Center Hospital, Saitama, Japan; Department of Surgery, Oita Prefectural Hospital, Oita, Japan

**Keywords:** pyrimidine nucleoside phosphorylase, oesophagus, carcinoma, human, reverse transcription polymerase chain reaction

## Abstract

To clarify the significance of the expression of pyrimidine nucleoside phosphorylase (PyNPase) mRNA as a predictive factor for the prognosis of patients with oesophageal carcinoma, the PyNPase mRNA in the tumours and normal tissues from 55 resected cases of oesophageal carcinoma was examined by a reverse transcription polymerase chain reaction (RT-PCR). As a result, a positive correlation was observed between the tumour/normal (T/N) ratio of the expression of PyNPase mRNA by RT-PCR and that of the enzyme activity of PyNPase based on the findings of an enzyme linked immunosolvent assay (*r* = 0.594, *P* = 0.009). The T/N ratio of the expression of PyNPase mRNA was significantly higher in the cases with lymph vessel invasion (*P* = 0.013), lymph node metastasis (*P* = 0.0016), and an advanced stage of the disease (*P* = 0.021) than those without these factors. The patients with a higher T/N ratio of PyNPase mRNA showed significantly worse prognosis than those with a lower T/N ratio (*P* = 0.023 with log-rank tests). A multivariate analysis for the cumulative survival rates revealed that a high T/N ratio of the expression of PyNPase mRNA was independently related to a poor prognosis. These findings suggested that the determination of PyNPase mRNA by RT-PCR thus appears to be a new useful parameter for identifying both a poor prognosis and a highly malignant potential of oesophageal carcinoma. © 1999 Cancer Research Campaign


*British Journal of Cancer* (1999) **79**(3/4), 565–569
© 1999 Cancer Research Campaign
Article no. bjoc.1998.0089

### Expression of pyrimidine nucleoside phosphorylase mRNA plays an important role in the prognosis of patients with oesophageal cancer

**M Yamagata[1], M Mori[1], K Mimori[1], K-I Mafune[2], Y Tanaka[3], H Ueo[4] and T Akiyoshi[1]**

[1]Department of Surgery, Medical Institute of Bioregulation, Kyushu University, Beppu, Japan; [2]Department of Surgery, Tokyo University Hospital, Tokyo, Japan; [3]Department of Surgery, Suita Cancer Center Hospital, Saitama, Japan; [4]Department of Surgery, Oita Prefectural Hospital, Oita, Japan

**Summary** To clarify the significance of the expression of pyrimidine nucleoside phosphorylase (PyNPase) mRNA as a predictive factor for the prognosis of patients with oesophageal carcinoma, the PyNPase mRNA in the tumours and normal tissues from 55 resected cases of oesophageal carcinoma was examined by a reverse transcription polymerase chain reaction (RT-PCR). As a result, a positive correlation was observed between the tumour/normal (T/N) ratio of the expression of PyNPase mRNA by RT-PCR and that of the enzyme activity of PyNPase based on the findings of an enzyme linked immunosolvent assay ($r = 0.594$, $P = 0.009$). The T/N ratio of the expression of PyNPase mRNA was significantly higher in the cases with lymph vessel invasion ($P = 0.013$), lymph node metastasis ($P = 0.0016$), and an advanced stage of the disease ($P = 0.021$) than those without these factors. The patients with a higher T/N ratio of PyNPase mRNA showed significantly worse prognosis than those with a lower T/N ratio ($P = 0.023$ with log-rank tests). A multivariate analysis for the cumulative survival rates revealed that a high T/N ratio of the expression of PyNPase mRNA was independently related to a poor prognosis. These findings suggested that the determination of PyNPase mRNA by RT-PCR thus appears to be a new useful parameter for identifying both a poor prognosis and a highly malignant potential of oesophageal carcinoma.

**Keywords:** pyrimidine nucleoside phosphorylase; oesophagus; carcinoma; human; reverse transcription polymerase chain reaction

Pyrimidine nucleoside phosphorylase (PyNPase), distributed mainly as thymidine phosphorylase in humans (Kono et al, 1983), is an enzyme involved in the salvage pathway of pyrimidine nucleotide synthesis (Weber, 1983). 5′-Deoxy-5-fluorouridine (5′-DFUR), a prodrug of 5-fluorouracil (5-FU), is converted to 5-FU by this enzyme (Ishitsuka et al, 1980; Eda et al, 1993). As a result, PyNPase plays an important role in the expression of the anti-tumour activity of 5-DFUR. In contrast, PyNPase has also been recently shown to be identical to a potent angiogenic factor, platelet-derived endothelial cell growth factor (PD-ECGF), which is thought to influence angiogenesis in tumour tissue (Ishikawa et al, 1989; Furukawa et al, 1992).

Previous studies have suggested the expression of PyNPase to be higher in malignant tissue than in normal tissue in several kinds of carcinomas, such as lung carcinomas, colorectal carcinomas, gastric carcinomas and so on (Nio et al, 1992; Luccioni et al, 1994; Takebayashi et al, 1996*a–c*; Giatromanolaki et al, 1997). The high expressions of PyNPase have also been revealed to be related to a high invasive potential or poor prognosis in carcinomas, including colorectal carcinoma, gastric carcinoma, renal cell carcinoma and bladder carcinoma (Takebayashi et al, 1996*a,b*; Imazono et al, 1997; Kubota et al 1997; Mimori et al, 1997). However, there have been few reports concerning the expression of PyNPase in oesophageal carcinoma. Thus, the clinical significance of higher PyNPase activity in oesophageal carcinoma has yet to be clarified.

Because the reverse transcription polymerase chain reaction (RT-PCR) method can amplify a target mRNA from a small amount of sample such as that obtained from a few biopsy specimens (Hirokoshi et al, 1992), this assay method is thus preferable to an enzyme assay which needs large samples of more than 100 mg (Eda et al, 1993). Mimori et al (1997) showed a positive correlation to exist between the expression of PyNPase mRNA by RT-PCR and the enzyme activity of PyNPase in colorectal carcinoma. The aim of the present study was, thus, to confirm the correlation between PyNPase mRNA and the enzyme level of PyNPase in oesophageal carcinoma, and to also demonstrate the clinical significance or usefulness of PyNPase mRNA expression in oesophageal carcinoma.

### MATERIAL AND METHODS

### Clinical samples

Fifty-five cases of oesophageal carcinoma were evaluated for this study. They consisted of 50 male and five female patients with an average age of 62.0 years (range 40–82). All patients underwent curative resection. Eleven patients underwent preoperative chemotherapy using 5-FU, cisplatin, etc. The tumours were located in the middle oesophagus ($n = 32$), in the upper oesophagus ($n = 4$) or in the lower oesophagus ($n = 19$) respectively. The stage of oesophageal cancer was determined pathologically according to the guidelines of the Japanese Society for Esophageal Diseases

*Received 10 December 1997*
*Revised 6 May 1998*
*Accepted 10 July 1998*

*Correspondence to:* M Yamagata, Department of Surgery, Medical Institute of Bioregulation, Kyushu University, 4546 Tsurumibaru, Beppu 874, Japan

(Japanese Society for Esophageal Diseases, 1992). The numbers of stage 1, 2, 3 and 4 patients were four, six, 38 and seven respectively.

### RNA preparation from tissue specimens

All specimens were taken from fresh tumour tissue, and the adjacent normal tissue at a distance of 5 cm from the tumour margin. Both the tumour and normal tissue specimens were confirmed by a histological examination. All the samples were immediately stored at –80°C until used in the following assays. The total RNA was then extracted according to a method previously described of acid guanidinium thiocyanate/phenol/chloroform extraction (Chomczynski and Sacci, 1987). All the samples were treated in Eppendorf tubes (Eppendorf, Germany).

### Reverse transcription

The total RNA was reverse transcribed using random hexamer primers into the complementary DNA (cDNA) as described previously (Mori et al, 1995, 1996).

### Oligonucleotide primer of PyNPase and GAPDH and semiquantative detection of mRNA

The preparation of oligonucleotide primers was performed with an Applied Biosynthesis 394 PCR-Mate DNA Synthesizer. All PCR primers were selected to span the introns to detect specific mRNA sequences. The primer sequence of PyNPase has also been described previously (Mimori et al, 1997). The primer amplified the 464-bp fragment of PyNPase cDNA. The amplified 450-bp fragment of glyceraldehyde-3-phosphate-dehydrogenase (GAPDH) cDNA was used as an internal control. An analysis of the semiquantative PCR of the PyNPase gene was also carried out. The most suitable number of PCR cycles for amplification of both GAPDH and PyNPase was 26 in the linear range of the curve, as previously determined using cultured carcinoma cell lines (Mimori et al, 1997).

### Polymerase chain reaction

The amplication of PyNPase cDNA was carried out in a total volume of 25 µl, which included 10× PCR buffer [100 mM tris-HCl (pH 8.3), 500 mM potassium chloride, 15 mM magnesium chloride 1% Triton X-100], 25 mM dNTP (mixed dATP, dCTP, dGTP and dTTP, each 100 mM), 15 mM each PyNPase primer and 1 unit of *Taq* DNA polymerase (Promega, Madison, WI, USA). The downstream primer was end labelled with [$\gamma$-$^{32}$P]ATP using T4 polynucleotide kinase (Takara, Japan). The reactions were subjected to 1 min at 94°C, 2 min at 60°C and 2 min at 72°C. The amplified DNA fragment was then electrophoresed on 1.5% agarose gel containing ethidium bromide with a DNA molecule weight marker for comparison. The PCR conditions for amplification of GAPDH have been described previously (Mimori et al, 1997).

### Comparison between the level of PyNPase and the semiquantitation of mRNA

Eighteen samples of the 55 cases were examined for the PyNPase levels using an enzyme-linked immunosolvent assay (ELISA), as described by Nishida et al, 1996). In these 18 cases, the enzyme levels indicated by ELISA was compared with the expression of PyNPase mRNA by RT-PCR.

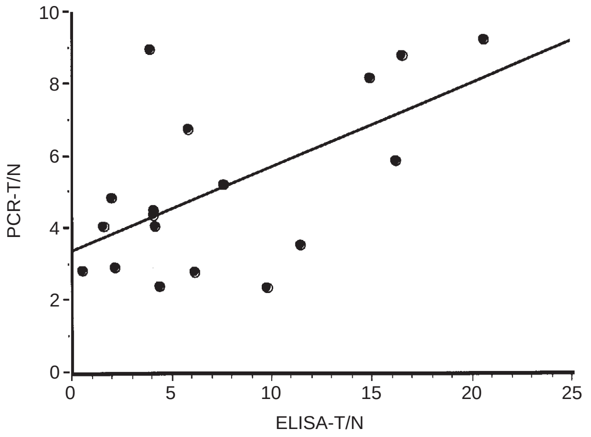

**Figure 1** The relationship between the tumour/normal (T/N) ratio of the PyNPase activity as determined by an enzyme-linked immunosolvent assay (ELISA-T/N) and the T/N ratio of expression of PyNPase mRNA by a reverse transcription polymerase chain reaction (PCR-T/N) was plotted ($n$ = 18). A positive correlation was observed between the two assays ($r$ = 0.594, $P$ = 0.009)

### Clinical evaluation of PyNPase mRNA

The clinicopathological factors were examined for their tumour/normal (T/N) ratio of PyNPase mRNA. The stage of oesophageal cancer was determined pathologically according to the guidelines of the Japanese Society for Esophageal Diseases (Japanese Society for Esophageal Diseases, 1992). The cumulative survival rates after surgical resections were compared using such variables as age, sex, location of the tumours, depth of tumour invasion, the histological type, lymph node metastasis, blood vessel invasion, lymph vessel invasion, stage, and the T/N ratio of PyNPase mRNA. All the significant variables obtained by a univariate analysis were then put into the multivariate regression analysis to identify any independent variables closely related to the survival rate after operation.

### Statistics

The data were expressed as the means ± s.d. Students' *t*-test was used to determine the statistical significance for univariate analyses. The survival rates were analysed by the Kaplan–Meier method (Kaplan and Meier, 1958). A log-rank test (Petro and Pike, 1973) was used to determine the statistically significant differences between the groups using a univariate analysis, and Cox's proportional hazard model (Cox, 1972) was used for the multivariate regression analysis by the Statview 4.5 statistical software (Abacus Concept, CA, USA).

### RESULTS

### PyNPase mRNA expression in oesophageal cancer

PyNPase mRNA was detected in all examined cases with oesophageal carcinoma. All samples were almost equivalent regarding the expression of GAPDH as an internal control. The T/N ratio for PyNPase expression was calculated and ranged from 0.51 to 17.76, with an average of 5.83. The median of the T/N ratio was 5.10.

© Cancer Research Campaign 1999

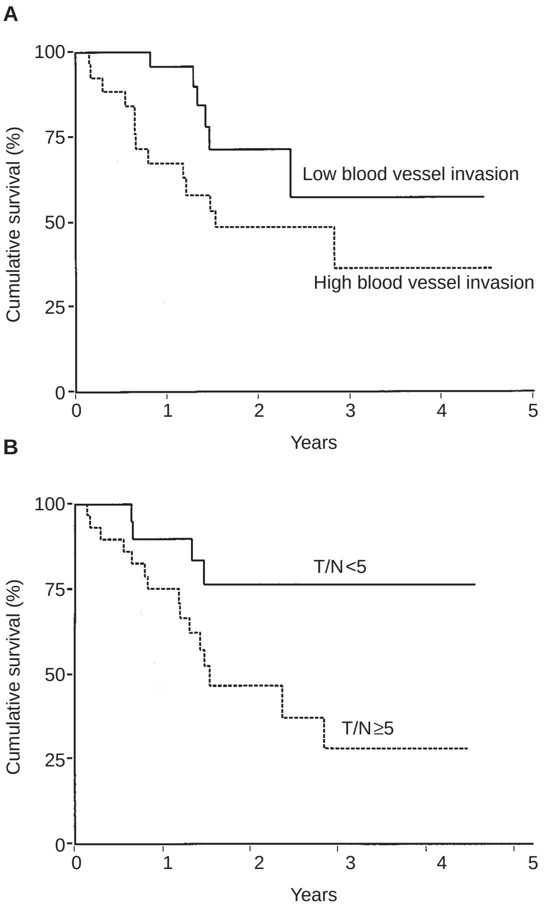

**Figure 2** The cumulative survival rate after resection of oesophageal carcinoma. (**A**) Fifty-five patients were divided into two groups according to the degree of blood vessel invasion defined histologically according to the guidelines of the Japanese Society for Esophageal Diseases (Japanese Society for Esophageal Diseases, 1992); low, $v_{0,1}$ (none or minimally invaded); and high, $v_{2,3}$ (moderately or highly invaded to the blood vessel). The patients in the former group survived significantly longer than those in the latter groups ($P = 0.044$ according to the log-rank tests). (**B**) Fifty-five patients were divided into two groups according to the tumour/normal (T/N) ratio of expression of PyNPase mRNA; T/N < 5 and T/N ≥ 5. The patients in the former group survived significantly longer than those in the latter group ($P = 0.023$ according to the logrank tests)

### Comparison between the PyNPase activity and the semiquantitation of mRNA

The T/N ratio of PyNPase activity using ELISA ranged from 0.49 to 20.48, with an average of 6.52. A positive correlation was, thus, observed between the T/N ratio of the PyNPase levels and the expression of PyNPase mRNA ($r = 0.594$, $P = 0.009$) (Figure 1).

### Clinicopathological characteristics

Table 1 shows the relationship between the clinicopathological factors of the patients and the T/N ratio of PyNPase mRNA. There was no significant difference in the T/N ratio of PyNPase mRNA in terms of the location of the tumour, the depth of tumour invasion or the histological type. Patients with lymph node metastasis were, thus, found to have a significantly higher T/N ratio of PyNPase mRNA than those without node metastasis ($P = 0.016$). The T/N ratio in patients with microscopic lymph vessel invasion was also significantly higher than that in patients without lymph vessel invasion ($P = 0.013$). A more advanced stage of the disease (stage 3 or 4) disclosed a higher T/N ratio ($P = 0.021$).

**Table 1** Relationship between various clinicopathological factors and the tumour/normal ratio of the pyrimidine nucleoside phosphorylase mRNA in oesophageal carcinoma

| Clinicopathological variables | Number of patients | T/N ratio of PyNPase mRNA[a] | *P*-value |
|---|---|---|---|
| Location of tumour | | | n.s. |
| Upper oesophagus | 19 | 5.80 ± 2.51 | |
| Middle oesophagus | 32 | 6.01 ± 3.64 | |
| Lower oesophagus | 4 | 4.86 ± 1.85 | |
| Depth of tumour invasion | | | n.s. |
| Within the adventitia | 37 | 4.96 ± 2.01 | |
| Beyond the adventitia | 18 | 6.29 ± 3.53 | |
| Histological type[b] | | | n.s. |
| Well differentiated | 15 | 5.36 ± 2.06 | |
| Moderately differentiated | 25 | 6.66 ± 3.92 | |
| Poorly differentiated | 11 | 5.33 ± 2.61 | |
| Unclassified or others | 4 | 4.38 ± 1.99 | |
| Lymph node metastasis | | | 0.016 |
| Absent | 9 | 3.55 ± 1.89 | |
| Present | 46 | 6.30 ± 3.17 | |
| Blood vessel invasion | | | n.s. |
| Absent | 13 | 5.04 ± 2.65 | |
| Present | 42 | 6.11 ± 3.29 | |
| Lymph vessel invasion | | | 0.013 |
| Absent | 9 | 3.51 ± 1.91 | |
| Present | 46 | 6.31 ± 3.16 | |
| Stage of disease[c] | | | 0.021 |
| 1, 2 | 10 | 3.80 ± 1.74 | |
| 3, 4 | 45 | 6.31 ± 3.23 | |

[a]Values are the means ± standard deviation. [b]All were squamous carcinoma other than unclassified or others which contained small cell carcinoma and adenocarcinoma. [c]Stage of disease was determined according to the guidelines of the Japanese Society for Esophageal Diseases (Japanese Society for Esophageal Diseases, 1992). n.s., no significant difference.

**Table 2** Results of a multivariate analysis using Cox's proportional hazards model

| Variable | Coefficient | s.e. | Relative risk | *P*-value |
|---|---|---|---|---|
| PyNPase mRNA ≥5 | 1.319 | 0.566 | 5.425 | 0.020 |
| High blood vessel invasion[a] | 1.021 | 0.497 | 4.226 | 0.040 |

[a]High blood vessel invasion was histologically determined as $v_{2,3}$ (moderately or highly invaded to the blood vessel) according to the guidelines of the Japanese Society for Esophageal Diseases (Japanese Society for Esophageal Diseases, 1992).

### Survival after a resection of oesophageal carcinoma

Fifty-five patients with oesophageal cancer were divided into two groups in association with the T/N ratio of PyNPase mRNA; one with a T/N ratio <5 and the other with a T/N ratio ≥ 5. Among the factors examined by a univariate analysis to identify the prognostic factors for the cumulative survival rate after resection, a high degree of blood vessel invasion and a high T/N ratio of PyNPase mRNA were both revealed to be poor prognostic factors. Figure

*© Cancer Research Campaign 1999*

2A and B shows the cumulative survival rates of the two groups divided by the degree of blood vessel invasion and the two groups divided by the value of the T/N ratio of PyNPase mRNA respectively. Patients without or with minimal blood vessel invasion had a better survival rate than those with higher blood vessel invasion ($P = 0.044$). Patients with a T/N ratio of PyNPase mRNA below 5 had a better survival rate than those with a higher T/N ratio ($P = 0.023$). There was a significant difference in lymph node metastasis between the two groups of T/N ratio of PyNPase mRNA ($P = 0.033$). Table 2 shows the results of the multivariate regression analysis for prognosis using the Cox's propotional hazard model. Both blood vessel invasion and the T/N ratio of PyNPase mRNA were confirmed to be independent prognostic factors.

### DISCUSSION

PyNPase is known to be involved in the invasion and metastasis of several kinds of tumours. In the present study, the cases with lymph node metastasis and/or lymph vessel invasion of the cancer disclosed a high T/N ratio of PyNPase mRNA expression in oesophageal cancer. This finding is consistent with previous reports in which malignancies that had high expression of PyNPase demonstrated a highly aggressive behaviour (Maeda et al, 1996; Takebayashi et al, 1996*a*,*b*; Tanigawa et al, 1996).

Thymidine phosphorylase, equivalent to PyNPase in humans, has been shown to be identical to a potent angiogenic factor, PD-ECGF (Ishikawa et al, 1989; Furukawa et al, 1992). Although the precise mechanism of angiogenesis by PyNPase remains unclear, previous reports have demonstrated the expression of PyNPase to be significantly associated with the intratumoral microvessel counts (Takebayashi et al, 1996*a*; Tanigawa et al, 1996; Imazono et al, 1997). Angiogenesis is generally known to play an important role in the early phase of carcinogenesis. In this study, the number of the patients in early stage was not sufficient to evaluate the role of PyNPase mRNA at an early phase of oesophageal carcinoma. In all examined patients, the expression of PyNPase mRNA did not correlate with the degree of vascular invasion, however it did correlate with both lymph vessel invasion and lymph node metastasis. As a result, the expression of PyNPase in oesophageal carcinoma might, thus, be at least involved in the further invasion and metastasis of the carcinoma. For further tumour growth and the development of tumour metastasis, other functions of PyNPase besides angiogenesis may also play an important role. Another possible mechanism may be the growth-promoting activity of the PyNPase/PD-ECGF (Ishikawa et al, 1989).

In addition, we also demonstrated that a higher expression of PyNPase mRNA in malignant tissue than in normal tissue resulted in a poor survival rate in the patients with oesophageal cancer. This fact may be mostly due to a positive correlation between the high expression of PyNPase mRNA and an advanced clinical stage. Lymph node metastasis contributes to the advancement of clinical stage (Sugimachi et al, 1994; Mori et al, 1997). A multivariate analysis for the cumulative survival rates revealed that the higher T/N ratio of the expression of PyNPase mRNA was independently related to a poor prognosis. This finding indicates that a high T/N ratio of PyNPase mRNA may not only be correlated with tumour aggressiveness, but may also be a useful predictive factor for a poor prognosis in patients with oesophageal cancer.

An enzyme assay for PyNPase activity using high-performance liquid chromatography (HPLC) requires a large amount of sample of more than 100 mg (Eda et al, 1993), whereas, in contrast, the RT-PCR method for detecting mRNA can be carried out using a very small sample including biopsy specimens (Hirokoshi et al, 1992; Mimori et al, 1997). Nishida et al (1996) suggested that there was good correlation between the enzyme levels of PyNPase measured by ELISA and the enzyme activity measured by HPLC. Our present study demonstrated a positive correlation between the PyNPase levels determined by the ELISA assay and the expression of PyNPase mRNA regarding the T/N ratio in oesophageal cancer. Therefore, the RT-PCR method allows us to determine the PyNPase status preoperatively from biopsy specimens.

The preoperative evaluation of the PyNPase condition of tumours is important, not only for determining the appropriate operative procedures but also for selecting the optimal anti-cancer drugs for adjutant chemotherapy. An extended resection may be necessary for patients with a probable poor prognosis predictable by the expression of PyNPase mRNA. An anti-cancer drug, 5′-DFUR, is a prodrug of 5-FU, and is converted to 5-FU by PyNPase (Ishitsuka et al, 1980; Eda et al, 1993). In fact, the anti-tumour effect of 5′-DFUR depends on the sensitivity of the tumour tissue to 5-FU. 5′-DFUR may be effective on carcinomas with higher activity of PyNPase and with sensitivity to 5-FU because of the possible selective cytotoxicity against tumour tissue of this anti-tumour drug, with a lower toxicity against normal tissue.

### REFERENCES

Chomczynski P and Sacci N (1987) Single-step method of RNA isolation by acid guanidium thiocyanate–phenol chloroform extraction. *Anal Biochem* **162**: 156–159

Cox DR (1972) Regression models and life tables. *J R Stat Ser B* **34**: 187–220

Eda H, Fujimoto K, Watanabe S, Ura M, Hino A, Tanaka Y, Wada K and Ishitsuka H (1993) Cytokines induce thymidine phosphorylase expression in tumor cells and make them more susceptible to 5′-deoxy-5-fluorouridine. *Cancer Chemother Pharmacol* **32**: 333–338

Furukawa T, Yoshimura A, Sumizawa T, Haraguchi M and Akiyama S (1992) Angiogenic factor. *Nature* **356**: 668

Giatromanolaki A, Koukourakis MI, Comley M, Kaklamanis L, Turley H, O'Byrne K, Harris AL and Gatter KC (1997) Platelet-derived endothelial cell growth factor (thymidine phosphorylase) expression in lung cancer. *J Pathol* **181**: 196–199

Hirokoshi T, Danenberg KD, Stadbauer THW, Volkenandt M, Shea LCC, Aigner K, Gustavsson B, Leichman L, Frosing R, Ray M, Gibson NW, Spears CP and Dannanberg PV (1992) Quantitation of thymidylate synthetase, dihydrofolate reductase, and DT-diaphorase gene expression in human tumors using the polymerase chain reaction. *Cancer Res* **52**: 108–116

Imazono Y, Takebayoshi Y, Nishiyama K, Akiba S, Miyadera K, Yamada Y, Akiyama S and Ohi Y (1997) Correlation between thymidine phosphorylase expression and prognosis in human renal cell carcinoma. *J Clin Oncol* **15**: 2570–2578

Ishikawa F, Miyazono K, Hellman U, Drexler H, Wernstedt C, Hagiwara K, Usuki K, Takaku F, Risau W and Helden CH (1989) Identification of angiogenic activity and the cloning and expression of platelet-derived endothelial cell growth factor. *Nature* **338**: 557–562

Ishitsuka H, Miwa M, Takemoto K, Fukuoka K, Itoga A and Maruyama HB (1980) Role of uridine phosphorylase for antitumor activity of 5′-deoxy-5-fluorouridine. *GANN* **71**: 112–123

Japanese Society for Esophageal Diseases (1992) *Guidelines for the Clinical and Pathological Studies on Carcinoma of the Esophagus*. Kanehara: Tokyo

Kaplan EL and Meier P (1958) Nonparametric estimation from incomplete observations. *J Am Stat Assoc* **53**: 457–481

Kono A, Hara Y, Sugata S, Karube Y, Matsushima Y and Ishitsuka H (1983) Activation of 5′-deoxy-5-fluorouridine by thymidine phosphorylase in human tumors. *Chem Pharm Bull* **31**: 175–178

Kubota Y, Miura T, Moriyama M, Noguchi S, Matsuzaki J, Takabayashi S and Hosaka M (1997) Thymidine phosphorylase activity in human bladder cancer: difference between superficial and invasive cancer. *Clin Cancer Res* **3**: 973–976

 © Cancer Research Campaign 1999

Luccioni C, Beaumatin J, Bardot V and Lefrancois D (1994) Pyrimidine nucleotide metabolism in human colon carcinomas: comparison of normal tissues, primary tumors and xenografts. *Int J Cancer* **58**: 517–522

Maeda K, Chung YS, Ogawa Y, Takatsuka S, Kang SM, Ogawa M, Sawada T, Onoda N, Kato Y and Sowa M (1996) Thymidine phosphorylase/platelet-derived endothelial cell growth factor expression associated with hepatic metastasis in gastric carcinoma. *Br J Cancer* **73**: 884–888

Mimori K, Mori M, Shiraishi T, Haraguchi M, Ueo H and Akiyoshi T (1997) Clinical significance of pyrimidine nucleoside phosphorylase in colorectal carcinoma. *Int J Oncol* **10**: 493–496

Mori M, Mimori M, Inoue H, Barnard GF, Tsuji K and Nanbara S (1995) Detection of cancer micrometastasis in lymph nodes by reverse transcription-polymerase chain reaction. *Cancer Res* **55**: 3417–3420

Mori M, Mimori K, Ueo H, Karimine N, Barnard GF and Sugimachi K (1996) Molecular detection of circulating solid carcinoma cells in the peripheral blood: the concept of early systemic disease. *Int J Cancer* **68**: 739–743

Mori M, Mimori K, Shiraishi T, Ueo H, Sugimachi K and Akiyoshi T (1997) p27 expression and gastric carcinoma. *Nature Med* **3**: 593

Nio Y, Kimura H, Tsubono M, Tseng CC, Kawabata K, Masai Y, Hayashi H, Meyer C, Fukumoto M and Tobe T (1992) Antitumor activity of 5′-deoxy-5-fluorouridine in human digestive organ cancer xenografts and pyrimidine nucleoside phosphorylase activity in normal and neoplastic tissues from human digestive organs. *Anticancer Res* **12**: 1141–1146

Nishida M, Hino A, Matsumoto T, Yoshikubo T and Ishitsuka H (1996) Preparation of anti-human thymidine phosphorylase monoclonal antibodies useful for detecting the enzyme levels in tumor tissues. *Biol Pharm Bull* **19**: 1407–1411

Petro R and Pike MC (1973) Conservation of the approximation (O–$E^2$)/E in the logrank test for survival data on tumor incidence data. *Biometrics* **29**: 579–584

Sugimachi K, Watanabe M, Sadanaga N, Ikebe M, Kitamura K, Mori M and Kuwano H (1994) Recent advances in the diagnosis and surgical treatment of patients with carcinoma of the esophagus. *J Am Coll Surg* **178**: 363–368

Takebayashi Y, Akiyama S, Akiba S, Yamada K, Miyadera K, Sumizawa T, Yamada Y, Murata F and Aikou T (1996*a*) Clinicopathologic and prognostic significance of an angiogenic factor, thymidine phosphorylase, in human colorectal carcinoma. *J Natl Cancer Inst* **88**: 1110–1117

Takebayashi Y, Miyadera K, Akiyama S, Hokita S, Yamada K, Akiba S, Yamada Y, Sumizawa T and Aikou T (1996*b*) Expression of thymidine phosphorylase in human gastric carcinoma. *Jpn J Cancer Res* **87**: 288–295

Takebayashi Y, Yamada K, Miyadera K, Sumizawa T, Furukawa T, Kinoshita F, Aoki D, Okumura H, Yamada Y, Akiyama S and Aikou T (1996*c*) The activity and expression of thymidine phosphorylase in human solid tumours. *Eur J Cancer* **32A**: 1227–1232

Tanigawa N, Amaya H, Matsumura M, Katoh Y, Kitaoka A, Aotake T, Shimomatsuya T, Rosenwasser OA and Iki M (1996) Tumor angiogenesis and expression of thymidine phosphorylase/platelet derived endothelial cell growth factor in human gastric carcinoma. *Cancer Lett* **108**: 281–290

Weber G (1983) Biochemical strategy of cancer cells and the design of chemotherapy: GHA Clowes Memorial Lecture. *Cancer Res* **43**: 3466–3492

*© Cancer Research Campaign 1999*
